# Progress of Artificial Intelligence in Drug Synthesis and Prospect of Its Application in Nitrification of Energetic Materials

**DOI:** 10.3390/molecules28041900

**Published:** 2023-02-16

**Authors:** Bojun Tan, Jing Zhang, Chuan Xiao, Yingzhe Liu, Xiong Yang, Wei Wang, Yanan Li, Ning Liu

**Affiliations:** 1 Xi’an Modern Chemistry Research Institute, Xi’an 710065, China; 2 Academy of Ordnance Science, Beijing 100089, China

**Keywords:** energetic materials, nitrification process, artificial intelligence, nitrification safety

## Abstract

Artificial intelligence technology shows the advantages of improving efficiency, reducing costs, shortening time, reducing the number of staff on site and achieving precise operations, making impressive research progress in the fields of drug discovery and development, but there are few reports on application in energetic materials. This paper addresses the high safety risks in the current nitrification process of energetic materials, comprehensively analyses and summarizes the main safety risks and their control elements in the nitrification process, proposes possibilities and suggestions for using artificial intelligence technology to enhance the “essential safety” of the nitrification process in energetic materials, reviews the research progress of artificial intelligence in the field of drug synthesis, looks forward to the application prospects of artificial intelligence technology in the nitrification of energetic materials and provides support and guidance for the safe processing of nitrification in the propellants and explosives industry.

## 1. Introduction

Propellants and explosives are the power source of weapon systems and the important material basis for achieving long-range precision strikes and efficient destruction. Compounds with a high explosive performance, low susceptibility and good chemical and thermal stability are ideal high-performance energetic materials sought by researchers [1,2,3,4]. The development of high-performance energetic materials to replace poorly performing energetic materials (such as propellants) has far-reaching significance and implications [5,6,7]. With the development of energetic materials, a variety of novel energetic materials, synthesis theories and methods have emerged in recent years and new energetic material molecules are constantly constructed. Nitration reaction is a common and important chemical reaction for the synthesis of energetic material molecules [8]. Nitro-energetic materials prepared by nitration are the most important and common type of energetic materials and are the most widely used. In recent years, the development of nitro-energetic materials has become one of the most important standards for measuring the level of development of a country’s weaponry. It is estimated that approximately 90% of all single component explosives currently need to undergo the nitration step [9]. However, the nitration reaction process is a strongly exothermic reaction and most nitro compounds have flammable and explosive properties. Therefore, nitration reactions have a great fire and explosion risk and are prone to being uncontrolled, serving as a hazardous chemical process under the key supervision of various countries [10].

To achieve the powerful military objective of “can fight and win battles”, the military industry, especially the energetic material industry, has put forward higher requirements for safe production. However, the scale, frequency and consequences of various production accidents are also expanding with the expansion of the production scale and capacity of nitro compound manufacturers, causing serious casualties, property losses and environmental damage [11,12]. Behind this series of tragic accidents, people wonder why accidents occur so frequently in the production and use of nitro compounds and why the consequences are so serious. After reading the accident investigation reports, the reasons for such accidents caused by nitro-chemical reactions are summarized as follows: (1) Operator misuse, including abnormal temperature rise, fire and entry into the air. (2) Materials with a series of hazardous characteristics, such as low ignition point, low boiling point, high sensitivity and a wide range of explosive limits. (3) The dangerous characteristics of the nitration process itself, such as high temperature, non-homogeneous reactions with high heat build-up, violent reactions and high heat release. (4) the reaction products and byproducts after the nitration reaction are explosive and post-processing (such as filtration, drying and concentration) required after the termination of the reaction may lead to an explosion due to friction or impact [13,14,15].

Careful studies of accident investigation reports show that operational errors are the direct cause of almost all accidents. Many companies have long adopted intermittent nitrification, that is, a batch of reaction material is put into the reactor and then allowed to react for a certain time before being removed. Such intermittent nitrification reactors have certain limitations in mass and heat transfer efficiency to strictly control the temperature, which easily causes local heat accumulation in the reactor, resulting in a series of side reactions (such as multiple nitrification and oxidation) and even explosive accidents [16]. In addition, the acid drop of nitrification reaction takes up to 10 h or even 20 h, which severely limits the nitrification capacity and the development and application of downstream products [17]. In recent years, continuous nitrification technology (including kettle-type continuous nitrification [18], tower-type continuous nitrification [19], ring-type continuous nitrification [20], microchannel continuous nitrification [21], adiabatic continuous nitrification [22], etc.) has made great progress and its risk is far lower than intermittent and semi-intermittent processes [23], which overcomes the shortcomings of intermittent nitrification reactions. In the continuous nitrification production technology, raw materials and nitrification reagents are fed into the reactor continuously and simultaneously according to the set ratio, the nitrification reaction is completed through efficient mass transfer and rapid heat transfer and then the products are discharged continuously from the reactor. The continuous nitrification production technology changes the status quo of the low production capacity and high labor intensity of traditional intermittent nitrification and greatly improves labor productivity [24]. The safety hazard of heat accumulation during the production process of intermittent nitrification has been fundamentally solved [25]. However, there are still several problems in the continuous process: (1) the dangerous post-processing stages (e.g., extraction, filtration, etc.) still require “manual“ handling and are not truly an example of “human-machine isolation”; (2) the device is less compatible with the control system. The continuous nitrification production technology is not as convenient and economical as an intermittent operation mode for relatively small-scale chemical reactions [26]. In recent years, artificial intelligence has made impressive research progress in the fields of drug discovery and development, exhibiting the advantages of high synthetic efficiency, reduced costs, short time and less waste [27,28,29,30,31,32,33]. Therefore, with the introduction of pathbreaking artificial intelligence into the continuous nitrification process, the development of continuous nitrification unit reaction technology and the significant reduction in the number of on-site operators or even the absence of operators on site, only remote manipulation can greatly improve the degree of “intrinsic safety” [34,35,36,37] to achieve a true sense of “human-machine isolation”. Artificial intelligence technology has been applied in the field of organic synthesis, but it has not yet been reported in the field of energetic materials nitrification. In view of this, this paper reviewed the research progress of artificial intelligence technology in the field of drug organic synthesis to provide new ideas for the application of artificial intelligence technology in the nitration reaction of energetic materials.

## 2. Advances of Artificial Intelligence in Drug Discovery

**Figure 1:**
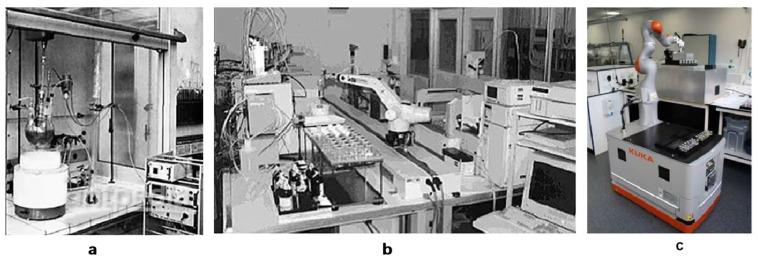
Evolution of the Automated Synthesis Machine platform: (**a**) 1970s; (**b**) 1999; (**c**) 2020. Copyright 2020, Springer Nature Liminted.

In 1950, Alan Turing, the “father of computing”, marked a milestone with his paper “Can Machines Think?” and brought a new discipline to mankind, artificial intelligence [38], which is now widely investigated by scientists. Artificial intelligence is an emerging technology that empowers robots with a degree of autonomous analysis and judgment, reducing the proportion of manual work in the overall workflow and the influence of human factors to ensure an efficient and reliable workflow. Machine learning models and algorithms serve as the core of AI, which are essentially machines programmed to learn patterns and laws in a data set based on a set of mathematical rules or statistical assumptions. Generally, machine learning aims to discover associations between features from a given data set to build predictive models and the output values can be binary responses, multicategory labels, or continuous values. A well-trained predictive model needs to have good generalization capability, i.e., it can more accurately predict samples outside the training set, including the more classical predictive models such as logistic regression models, decision tree models, Bayesian probability models, support vector machines, convolutional neural networks, etc. Artificial intelligence machines can be traced back as far as the 1960s and 1970s (Figure 1a). At that time, a single chemical reaction process was controlled by a computer that automatically performed operations, such as dosing, stirring, heating/cooling, refluxing, etc. With the rapid development of chips, computers and precision mechanics over the next 10–20 years, various functional modules (such as high-throughput screening of reaction conditions, purification of crude products and characterization of products) were gradually integrated into automated chemical synthesis systems, such as the one introduced in 1999 (Figure 1b). In the 21st century (Figure 1c), “deep learning” techniques were first applied in chemistry in the direction of small molecule design, including artificial intelligence for synthetic route planning and reverse molecular design. Researchers developed software with learning capabilities using various algorithms, such as Monte Carlo algorithms and genetic algorithms. When mechanical automation and artificial intelligence algorithms are developed to a certain extent, the combination of the two will give birth to “autonomous discovery systems” (i.e., algorithmic programs), which will automatically adjust experimental solutions based on experimental results and iterate until a preset target is obtained, and so automatic synthesis machines begin to show “intelligent” characteristics [39,40,41,42,43].

Artificial intelligence-assisted automatic synthesis machines are the current international research frontier, integrate big data science, artificial intelligence, chemical synthesis, automatic control, precision manufacturing and other disciplines, and have attracted great interest and attention from countries around the world. On the one hand, as a derivative of the information technology revolution, AI involves industrial manufacturing, transportation, people’s livelihood, energy and other fields, and serves as one of the technological high points seized upon by the major technological powers [44,45,46]. On the other hand, the continuous learning capability of AI based on massive data and the ability of intelligent exploration in unknown spaces effectively fits the current needs of synthetic engineering trial-and-error platforms and AI has great potential in combination with the design of nitro-synthesis systems for energy-containing compounds under complex and hazardous experimental conditions. Currently, this research focuses on drug synthesis and has made significant progress. The representative foreign work is listed in Table 1.

In the process of drug development and synthesis of natural products, there is a lack of raw materials for reactants and the test requires as few doses as possible. Moreover, it is difficult to realize the micro-quantization and automation of these reaction processes in the presence of solid or volatile solvents. Therefore, there is a great need for AI reaction screening systems to select the optimal reaction precursors and conditions to improve the efficiency of synthesis.

### 2.1. Artificial Intelligence High-Throughput Chemical Reaction Screening Platform

In 2015, Merck [47] developed a high-throughput chemical reaction screening platform, which combined microarray technology and low-resolution mass spectrometry technology to screen the substrates and reaction conditions of Buchwald-Hartwig coupled reactions at nano-molar magnitude. The efficiency can reach an amazing 1536 reactions per day. This technology promises to provide more powerful tools for high-throughput synthesis and greatly accelerate the process of drug discovery. The only drawback of this microplate-based system is that only the high boiling point polar solvent DMSO (dimethyl sulfoxide) can be used in the reaction and the reaction temperature is limited to room temperature, which limits the scope of the platform.

**Figure 2:**
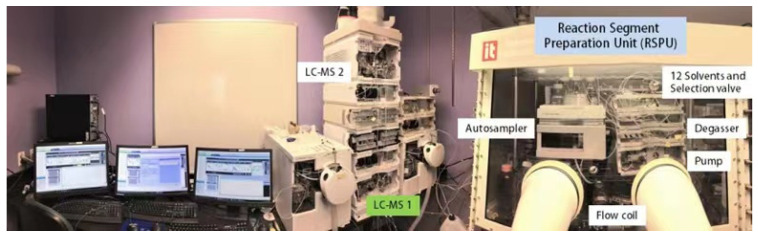
Pfizer’s automated high-throughput chemical reaction screening platform [48].

In 2018, Pfizer [48] developed a platform for automated high-throughput chemical reaction screening under different solvents, temperatures, pressures and other conditions. (Figure 2). All components of the platform are commercially available on the market. Based on the flow chemistry technology and ultra-high performance liquid chromatography-mass spectrometry technology, more than 1500 nanomolar Suzuki-Miyaura coupling reactions can be screened in one day and the synthesis of hundreds of micromolar orders can also be supported at the same time. The automation platform is mainly composed of several computer control units, a continuous flow chemical reaction device placed in the glove box and two sets of UPLC-MS devices that can be switched. The researchers first mixed the reactant, catalyst, ligand and base into solutions and placed them in two reagent bottles with 96 well plates (192 reagent bottles in total). The autosamplers were instructed by a computer to take 1 µL of the solution from each of the five vials and pump it into a mixing device. The mixed material (5 µL) was diluted with the reaction solvent (500 µL) and then flowed into the temperature-controlled micro-reactor for reaction. Finally, the mixture from the micro-reactor was separated by UPLC and analyzed online by UV-Vis spectroscopy and mass spectrometry. At the same time, the computer program feeds back instructions to the auto-sampler to prepare the next group of reaction samples, which will be detected by another UPLC-MS and the two UPLC-MS devices will detect alternately, the platform able to screen a group of reactions every 45 s. The flow rate, residence time, sampling, injection and reaction solvent switching in the reaction are all controlled by the computer. The most important feature of this method is that the reactant and reaction reagents are dissolved in advance by using a non-reactive solvent and the components can be fully mixed and evenly dispersed in the reaction solvent, that is, the reaction can be carried out in a homogeneous phase. In the past, when using flow chemistry technology to optimize reaction conditions, the reactants and reagents generally needed to be dissolved in the reaction solvent in advance. Once the reaction solvent was changed, the reactants and reagent solutions had to be reconfigured. In addition, to process optimization with expensive reagents on the nanomoles, the reaction platform can also enable product synthesis in the order of hundreds of micromoles. For example, under the optimal reaction conditions, 100 groups of Suzuki-Miyaura coupling reactions were performed continuously and each reaction was controlled at 3.2 µmol scale (the total reaction scale is 0.32 mmol) and 65 mg of compound can be obtained with a total separation yield of 59%. Surprisingly, when the reaction was scaled up using the intermittent reaction flask, the target product can be obtained in a yield of up to 79% by increasing the reaction concentration to 0.1 M. In contrast, the yield of compound was only 43% when the above reaction was amplified using a conventional flow chemical reactor. The researchers then turned their attention to the more challenging Suzuki-Miyaura coupling reaction of boric acid ester and bromoindole ketone. They completed the screening of 576 reaction conditions in 8 h and only consumed 50 mg of raw material 5. The optimal conditions were CataCXium A as catalyst, Et3N as base and THF/H_2_O (9/1) as solvent. On this basis, the reaction can be successfully scaled up to 0.41 mmol by intermittent reaction flask with a yield of 81% only by slightly adjusting the material ratio, reaction concentration and reaction time. Pfizer’s research opens up new avenues for high-throughput chemical reactions under different solvents and heating conditions. Combined with flow chemistry and UPLC-MS, 5760 nanomolar reactions can be evaluated within 4 days and more than 1500 reactions can be screened per day. Moreover, the target product can be directly synthesized in the order of hundreds of micromoles through batch multiple injections, which can quickly meet the requirements of subsequent possible biological activity tests. More importantly, the optimized reaction conditions can be enlarged without major changes in the traditional batch or flow reaction mode and the compound can be prepared in milligram order with excellent yield. This new technology can greatly accelerate the synthesis efficiency of complex compounds and will show broad application prospects in various fields including drug research and development.

**Figure 3:**
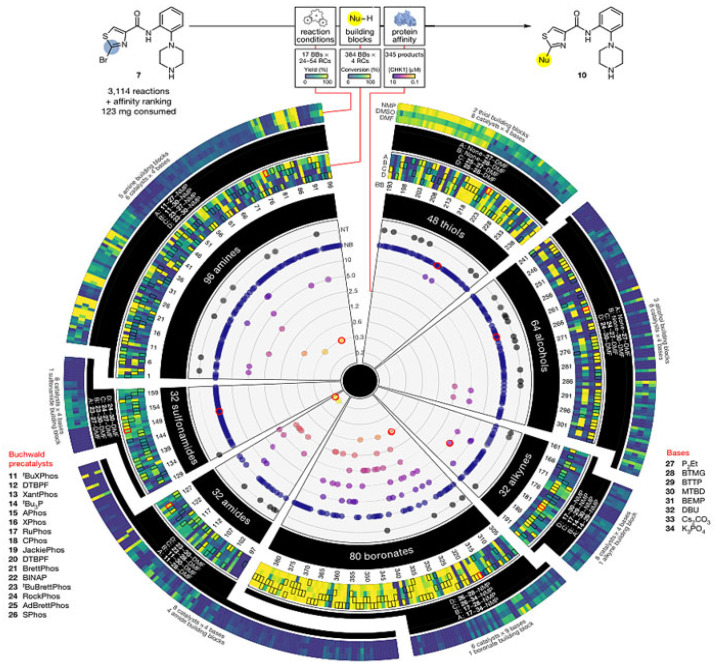
NanoSAR technology for high-throughput screening of CHK1 inhibitors and their synthesis conditions. Reprinted with permission from ref. [49]. Copyright 2018, Springer Nature.

In 2018, chemists at Merck [49] addressed the scarcity of reactants in the subsequent steps of drug development and natural product synthesis, the need for as few doses as possible in tests and the difficulty of micro-quantification and automation in the reaction process (Figure 3). A “massive” upgrade to their previously reported automated high-throughput reaction screening platform added an important feature, i.e., compound bioactivity testing. Specifically, the affinity of the reaction product to the target protein was determined directly using label-free affinity-selection mass spectrometry (ASMS) after the completion of the high-throughput nanomolar (less than 0.05 mg of substrate consumed per reaction) conjugate reaction. It also avoided the time-consuming purification steps of conventional methods and avoided the synthesis and isolation of a large number of candidate compounds without affinity activity. They named their new technique NanoSAR (Nanoscale synthesis and affinity Ranking) for the integration of high-throughput nanomolar synthesis and affinity screening ranking. The results showed that this technology could help quickly screen out the products and synthesis conditions of CHK1 that could inhibit the target protein kinase from 3114 nanomolar coupling reactions, which greatly accelerated the speed of lead drug discovery and reduced the amount of scarce raw materials. Moreover, some potentially active compounds discovered by NanoSAR technology are completely undetectable by conventional methods. Merck research provided a powerful tool for drug discovery. Combining automated high-throughput nanomolar chemical reactions with affinity selection-mass spectrometry, thousands of nanomolar reactions can be evaluated in the shortest time with the least amount of raw materials, screen reaction substrates and reaction conditions. Moreover, a large number of product mixtures can be screened and sorted for the affinity of target proteins without separation and purification, so as to predict the bioactivity of these products and thus guide the subsequent synthesis, purification, bioactivity testing and so on. There is no doubt that NanoSAR technology, which integrates high-throughput reaction screening with affinity screening, can help medicinal chemists to avoid the synthesis and purification of a large number of bioactive compounds and to screen compounds more comprehensively and quickly, thereby identifying the most potential targets and improving the “hit accuracy” of drug discovery.

In 2018, Professor Abigail Doyle et al. [50] began to use a powerful machine learning algorithm called Random Forest Algorithm; after receiving thousands of Buchwald-Hartwig coupling reaction data, this algorithm can accurately predict the yield of other Buchwald-Hartwig coupling reactions that have not been reported in the literature. If the data of this algorithm is greatly reduced to hundreds of reactions and the reaction outside the sample is predicted by this algorithm, the prediction results of this algorithm are still consistent with most of the experimental data. Professor Abigail Doyle believes that the accurate prediction performance of AI in this study is mainly due to the establishment of a super large high-quality learning database and, of course, the selection of reasonable chemical descriptors, which also needs the help of Spartan, a high-throughput reaction and chemical computing software.

In 2018, Professor Mark Waller et al. [51] reported a new artificial intelligence (AI) tool that can design molecular synthesis routes through self-learning of organic reactions. In this work, the research team used a previously developed Deep Neural Network system to automatically extract the chemical conversion rules from the 12.4 million single-step reactions in the Reaxys Database before 2015. After selection, only the “high-quality” rules that repeatedly appear in the reaction for more than a certain number of times were retained. Subsequently, they used three different neural networks combined with Monte Carlo Tree Search (MCTS) to form a new AI algorithm (3N-MCTS), which relies on automatically extracted rule data for training and deep learning. This new AI tool did not require chemists to input any rules, but could learn the chemical conversion rules by itself based on the reported single-step reaction and conducted rapid and efficient reverse synthesis analysis. This indicated that the new AI algorithm had exceeded the current algorithm in predicting molecular synthesis routes and was as reliable as human chemists. The greatest significance of this work was that AI tools would improve the success rate of synthetic chemistry, help drug discovery programs increase speed and efficiency and reduce costs. The new artificial intelligence (AI) tools reported above will undoubtedly greatly liberate the productivity in the field of automating small molecule synthesis. However, due to the lack of common standards, only individual reactions can be automated at present. All of these methods are continuous iterations based on a small number of strong robust reactions. Therefore, they cannot be programmed and unit operations cannot be reused, which greatly limits the development of the general field of synthetic automation. At present, based on the above problems, chemical synthesis automation platforms based on different strategies have been established. 

### 2.2. Modular Automatic Synthesis Machine Driven by Chemical Programming Language

**Figure 4:**
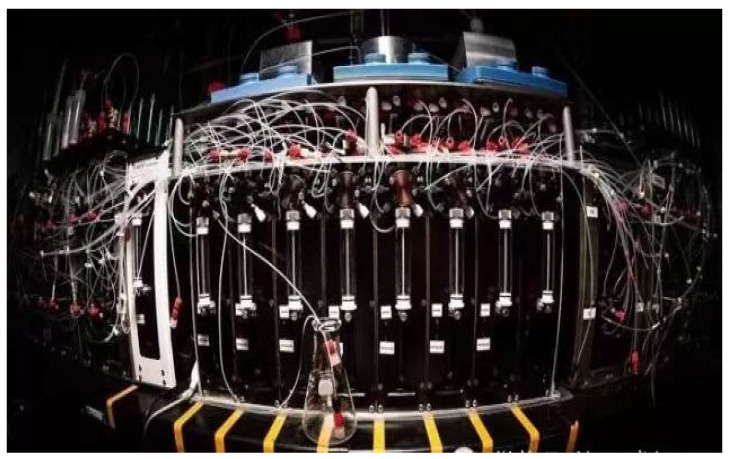
Model diagram of “super machine”. Adapted with permission from ref. [52]. Copyright 2015 The American Association for the Advancement of Science.

In 2018, Martin Burke et al. [52] designed a new “super machine”, which can select components from pre-prepared molecular building blocks and automatically complete the synthesis process according to the target molecular structure. The synthesis step of this “super machine” is to couple all the molecular blocks containing the same connecting group through a simple reaction and one molecular block after another reacts and connects in turn (Figure 4). The whole synthesis process is like playing Lego, which greatly simplifies many complex synthesis steps, and the simplification makes automation possible. Since this large number of molecular blocks have been commercialized, more small-molecule compounds can be synthesized automatically using this machine. For this strategy, they constructed a method of “capture-release” of intermediates: after each coupling reaction, the reaction intermediates were fixed on silica gel, the excess reactants and by-products were eluted and removed and then the reaction intermediates were released to enter the next reaction. Each reaction cycle included deprotection (D), coupling (C) and purification (P). This was repeated until the target molecule was synthesized through the linear precursor in series and then connected back to itself. The complex polycyclic structure could also be synthesized automatically. Professor Burke’s research team has used this “super machine” to synthesize 14 kinds of small molecules at the milligram scale. There is no doubt that this automated synthesis system will greatly accelerate the efficiency of small molecule synthesis and has great potential in a wide range of fields including drug research and development. Moreover, if this automatic synthesis method and “super machine” can be widely used in the field of organic synthesis, it will have a revolutionary impact on the existing concepts and technologies of organic chemistry. Some chemists have even commented that this technology may lead to “The End of Synthesis”.

**Figure 5:**
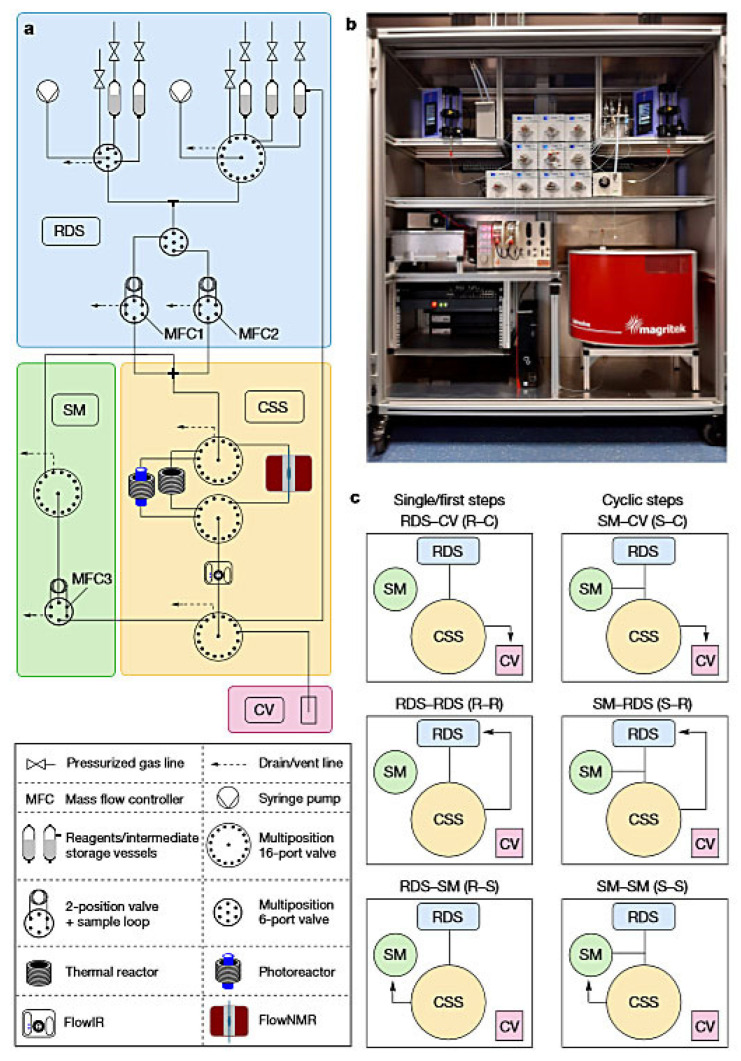
Radial synthesis equipment: (**a**–**c**). Reprinted with permission from ref. [54]. Copyright 2020, Springer Nature.

**Figure 6:**
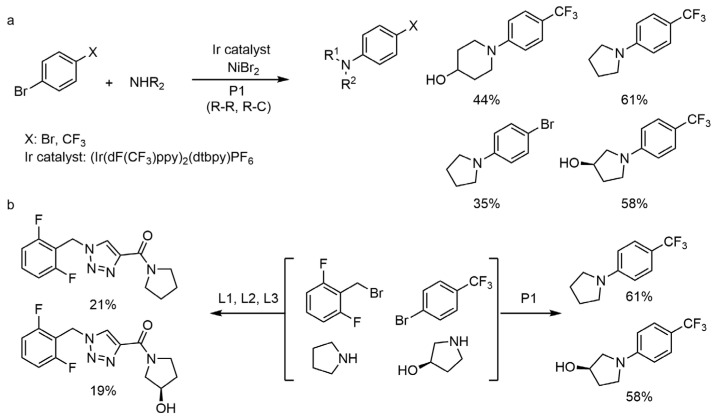
Modular expansion of system functions: (**a**,**b**). Adapted with permission from ref. [54]. Copyright 2020, Springer Nature.

Although great progress has been made in the field of robotics, the ability to “think before acting” is the robot’s weakness. In 2019, Professor Leroy Cronin et al. [53] found that robots can also have the “intuition” of human chemists (Figure 5). In order to prove this, they developed a new machine learning algorithm to control the organic synthetic robot, which can independently “think” after completing the experiment, so as to understand and decide how to proceed next. The automatic process was realized in a modular robot platform, which would run a chemical programming language to control the assembly of related molecules. Even users who do not know programming knowledge can easily code. Chemputer was used to produce three high-quality medicinal compounds without any human intervention: diphenhydramine hydrochloride, rufamide and sildenafil, with yield and purity comparable to that of artificial synthesis. The chemputer system was composed of different modules, including reaction module, internal filtration module (which can be heated or cooled), automatic liquid–liquid separation module and solvent evaporation module. The different processes of chemical synthesis were divided into graphical modules. The whole chemical synthesis process requires not only the chemputer software, but also some physical equipment: bottled reagents, round-bottom flasks, filtration and liquid–liquid separation devices, rotary evaporators and pipes, valves and pumps for transporting chemicals. The researchers input the synthesis methods and step instructions from the literature into the steps suitable for automation and the synthesis steps are converted into digital codes, which can be flexibly released between platforms without modification, thus greatly improving the repeatability and the synthesis rate of complex molecules. By developing control software to automatically clean and reuse the hardware modules in subsequent reaction steps, a multistep synthesis process from independent unit operation to combined complete synthesis was completed and finally used in laboratory scale organic synthesis. It is worth noting that chemputer still has many shortcomings to be overcome. For example, the synthesis routes of the three drug molecules are relatively short and the separation is relatively simple. Most of them only involve extraction and separation. However, it is difficult to achieve the separation effect of such high purity described by the researchers in the steps with many side reactions. In addition, analytical techniques such as LC-MS, NMR and IR are not mentioned, which means that these steps can only be repeated with the chemputer system after the final optimization (Figure 6).

In 2021, Dr. Kerry Gilmore et al. [54] gave a positive answer. They reported a new type of automatic radial synthesis equipment, which was used for automatic synthesis of small molecules and integrates the advantages of cyclic synthesis and linear synthesis. The core of the device was the Central Switching Station (CSS) unit with a series of continuous flow modules connected to it in a radial arrangement around it. This fully automatic equipment can perform linear synthesis and convergent synthesis and can effectively synthesize various types of small molecules, such as the anticonvulsant lufilamide and its derivatives, without time-consuming manual adjustment between different processes. This radial synthesis device was composed of four parts: solvent and reagent delivery system (RDS), transfer station (CSS), standby module (SM) and collection container (CV). The transfer station includes a series of independent reactors and online analytical instruments. In the process of radial synthesis, multiple single-step conversion or multiple steps of multi-step reaction were not carried out simultaneously, but occurred separately and sequentially. In this way, the reactor can be reused under different conditions and the residence time can be controlled as needed, regardless of any previous steps. This not only greatly reduced the amount of equipment required, but also avoided system reconfiguration when switching between different synthesis processes. In order to further explore the applicability of this radial synthesis device, the author used it to the synthesis of a lufilamide derivatives library and prepared a variety of derivatives with high yield. In addition, the radial synthesis device can be further expanded by adding only a photochemical module (420 nm LED photoreactor), which can perform a wider and more diverse chemical process without reconstructing the reaction system using the same reagent. They obtained lufilamide derivatives with good separation yield. The radial automatic synthesis instrument developed by Dr. Kerry Gilmore et al. can not only performed linear synthesis and aggregation synthesis, but also on-lined dilution for concentration and solvent screening and realized single-step or multi-step synthesis as required, without manual reconstruction of the instrument. In addition, the reactor can be reused at different temperatures and flow rates and the intermediates can be stored for aggregation synthesis and multi-step optimization. This instrument can be said to be the first truly universal automatic synthesis equipment.

### 2.3. AI Synthetic Robots with Thinking Functions to Explore New Reactions

**Figure 7:**
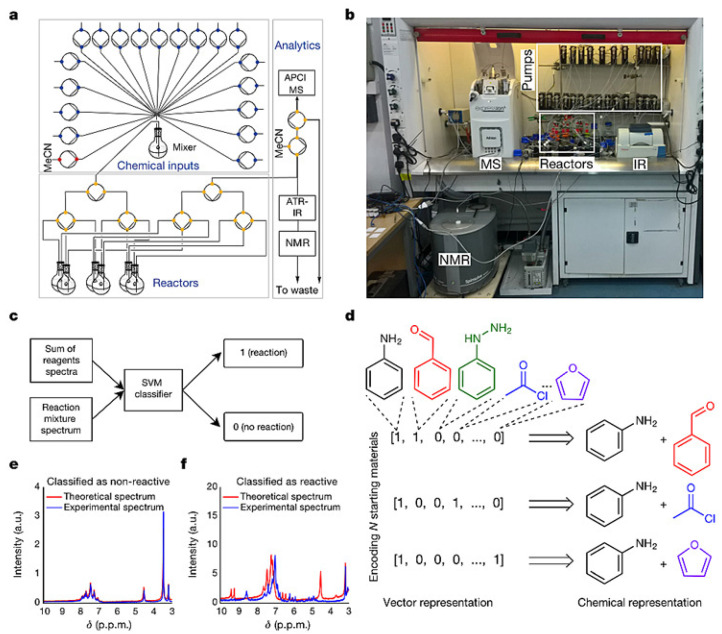
Synthetic robotic system with machine learning capabilities: (**a**–**f**). Reprinted with permission from ref. [55]. Copyright 2018, Macmillan Publisher Ltd., part of Springer Nature.

Leroy Cronin’s team [55] has developed a new machine learning algorithm to control organic synthesis robots, which is capable of completing chemical reactions, connecting analytical devices such as NMR, IR and MS for real-time feedback and adjusting reaction conditions based on algorithms generated from expert experience and autonomously exploring the reactivity of 1000 combinations of reactions. The robot can “think” independently after completing an experiment in order to figure out and decide what to do next, which gives it the ability to explore new chemical reactions and molecules and accurately predict the outcome of chemical reactions (Figure 7). The core components of this synthetic robotic system included a set of feedstock tanks containing chemicals and pressure pumps, which were responsible for feeding the reactants into six reaction vials that can be operated in parallel. When the reaction was complete, the mixture was sequentially fed into an infrared spectrometer, mass spectrometer and nuclear magnetic resonance instrument for testing and finally the support vector machine model was used to compare the fingerprints before and after the reaction to determine whether the raw materials produced chemical reactions and the degree of reactivity. Instead of extracting chemical information or having any chemical knowledge, the AI robot uses simple “digitized” data to describe each reaction and train machine learning algorithms. Specifically, the robot used binary coding for all variables throughout the experiment, which described each response as a vector of zeros and ones (similar to one-hot encoding), in order to train the machine learning algorithm. For example, in an experiment where the reaction conditions were fixed (that is, all reaction ingredients were variable), it defined the starting ingredients that occur as 1 and those that do not as 0. These simple numbers were formed into a vector containing information on all reactants used to characterize the reaction and were used as data on the input side of the machine learning. On the other hand, the support vector machine model determines whether a chemical reaction has occurred and classifies it by identifying changes in the spectrum before and after the reaction: if the reaction activity is high, then the result of that experiment will be defined as 1 and as 0 for the opposite as the data at the output of the machine learning. Then the robot used a linear discriminant analysis algorithm to learn from this ‘digital’ data, which is unrelated to the chemical structure, to look for patterns behind the reactions and to predict the outcome of unknown reactions with different variables, thus guiding the next experiment. In this study, the researchers wanted to quickly identify combinations of ingredients from 18 compounds that could produce chemical reactions, using uniform conditions for the reactions in order to reduce the workload and focusing only on two-component and three-component reactions, with about 1000 experiments. At first, the robot randomly selected 100 experiments for an initial trial, determined the results using an SVM model and then summarised the ‘digital’ data using the LDA algorithm. Then the AI predicted and gave feedback on the remaining experiments based on its self-learning knowledge, on the basis of which the robot prioritizes the 100 experiments it believes are most likely to produce a chemical reaction in order to start a second attempt. For every 100 experiments sampled, the robot automatically updated its database and used an algorithm to re-learn, reassessing the remaining reactions and continuing with 100 new attempts until either the specified number of experiments were completed or it determined that the remaining experiments would not produce a chemical reaction. The result was that the robot was able to select the more reactive combination of chemicals to explore from the remaining experiments each time and maintained an accuracy of over 80% in each prediction. By working in a “think twice before you act” mode like a human, the robot has also discovered some unprecedented types of reactions and molecules. As well as being able to explore new reactions like a human chemist, robots can also do something that is beyond the reach of chemists, such as predict the yield of chemical reactions. The researchers added a neural network algorithm to the robot, still using chemically unrelated ‘digital’ data to describe 5760 Suzuki-Miyaura coupling reactions and the neural network was able to learn from 3456 of these experiments in order to accurately predict the yield of the remaining 2304 Suzuki-Miyaura coupling reactions with a standard error of just 11%.

This level of success does not satisfy the chemists behind the robotic “chemists”, as the large amount of data often requires a large number of experiments to support them and, even with high-throughput automated reaction systems, this can still be resource intensive. Obviously, how to use a small amount of experimental data to quickly find high-yielding chemical reactions is the real winning strategy. After 576 coupling reactions were randomly selected for learning, the robot chemist selected the top 100 coupling experiments among the remaining reactions that were predicted to give high yields for testing. Preliminary results showed that the true average yield of these reactions was not high, at only 39% and the prediction error was large (27%). Nevertheless, after importing these 100 new data and re-learning the machine, the robot’s prediction level improved dramatically, achieving a true average yield of 85% for the top 100 quasi-high-yielding responses it selected from the remaining responses, with a prediction standard error of just 14%. As the data is updated and continuously learned, the robot is not only able to maintain a high level of performance in each subsequent prediction, but also always prioritizes the responses with relatively high true yields from the remaining experiments.

**Figure 8:**
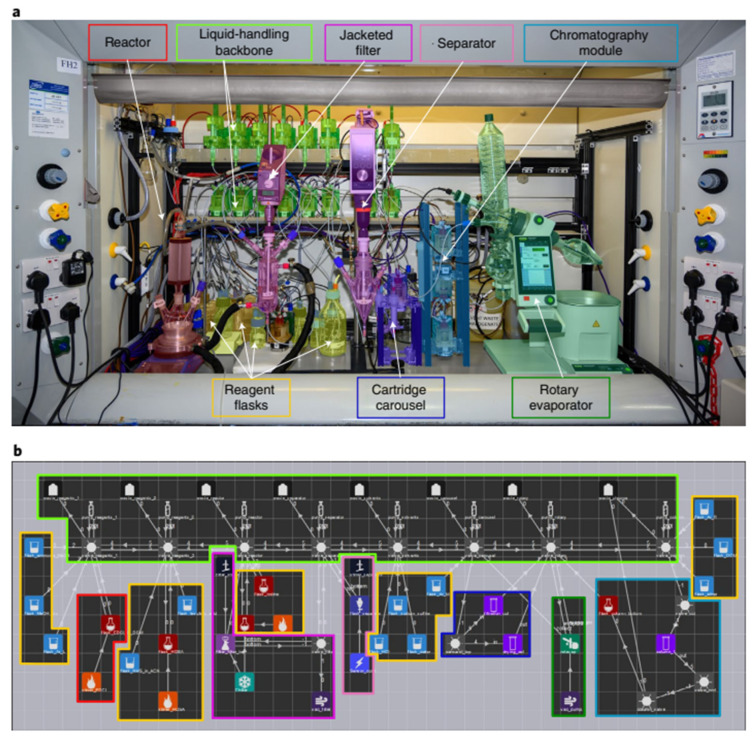
Implementation and structure of the automated synthesis platform: (**a**) hardware module for synthesizing the NHS-diazirine; (**b**) diagram of the corresponding software. Reprinted with permission from ref. [56]. Copyright 2020, Springer Nature Liminted.

However, most automated platforms require the use of bespoke hardware for each reaction, meaning that it is difficult to cascade multiple reactions on a single machine to automatically synthesize a target molecule. In 2021, Leroy Cronin’s group [56] demonstrated that Chemputer can be programmed to perform many different reactions in a unified system, including solid-phase peptide synthesis (SPPS), iterative cross-coupling (ICC) and the synthesis of unstable diazines (Figure 8). The focus is on the development of universal and modular hardware that can be automated using a software system. In the experiment, the authors’ system performed approximately 8500 operations, reusing only 22 different steps in 10 modules and the code was able to support 17 different reactions. Previously, Chemputer had demonstrated that a range of different molecules could be synthesized automatically on the same hardware. However, integrating the different existing automation strategies remains a major challenge. The authors argue that there is a need to develop a universal approach that makes the system programmable and modular, capable of unifying the various synthesis steps to enable the synthesis of almost any molecule that can be synthesized artificially. The authors show how the Chemputer system was developed to enable a single system to automatically perform iterative cross-coupling reactions based on MIDA-boronate, solid-phase peptide synthesis and the synthesis of the photo-crosslinker NHS-diazirine, which has important applications in biology and materials science. This work demonstrated that, through a modular approach, a single platform architecture can be used to unify the automated synthesis of different classes of molecules. 

Only minor modifications to the actual hardware example of the system are required to accommodate the different chemical reactions for these molecules. Compared to previous work on automated synthesis machines, the highlight of this research was the ability to perform then multiple sequential steps of different reactions on the same platform to obtain the final product and the prospect of establishing a uniform standard of experimental operation, solving the problem of experimental reproducibility. However, it is the process of figuring out the reaction conditions and crossing the columns that is undoubtedly the most time-consuming for organic chemists. The synthesis machine in this paper only seems to be suitable for reactions with complete synthesis conditions, which are then standardized and flowed. However, for the more time consuming and critical process of figuring out the conditions, it seemed to be powerless.

In the same way that human chemists act in experiments, robots can independently and autonomously explore new reactions and molecules in chemistry; in addition, they have the ability to accurately predict the outcome of chemical reactions. Exploring the chemical space in real time could help chemists to explore more and more useful molecules and reactions and can allow the drug development process to reduce costs, time and waste. There is no doubt that the advent of intelligent robots is making chemistry easier and will facilitate a new digital era in chemistry.

**Figure 9:**
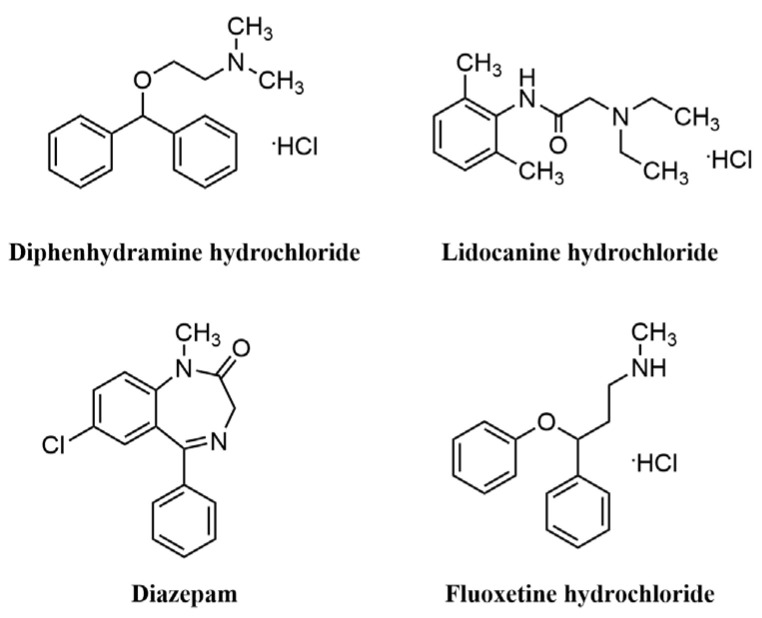
The structures of the drugs synthesized.

In 2019, Timothy F. Jamison et al. [57] made a breakthrough in the field of automated synthesis by developing a small automated diversity synthesis platform based on algorithms, flow chemistry and analytical techniques that can perform homogeneous or non-homogeneous catalytic reactions while being compatible with reactions involving C-C bonding/C-N coupling, Horner-Wadsworth-Emmons olefination, Paal-Knorr pyrrole synthesis, reductive amination, aromatic nucleophilic substitution, cycloaddition, photocatalysis and other different types of chemical reactions, end-to-end, from the optimization of reaction conditions to the synthesis and isolation of the target molecule (Figure 9). The system consists of an ‘upstream’ unit containing the reactor and a “downstream” module for precipitation, filtration, dissolution, crystallization and formulation. It also includes chemical analysis and calculation modules for process evaluation and control. To demonstrate the performance of the system, the team used it to produce oral and topical liquid formulations of the antihistamine Benadryl hydrochloride, the local anesthetic lidocaine hydrochloride, the sedative diazepam (Valium) and the antidepressant fluoxetine hydrochloride (Prozac or Sarafem). The researchers also hope to develop the capability to produce solid pill formulations. However, the drug structures synthesized by this platform are relatively simple and the continuous flow synthesis methods involved in this instrument may not yet be practical for drugs with more complex structures. The researchers will later optimize and upgrade the instrument platform system and expect the next version of the system to be a further 40% smaller and capable of synthesizing more complex drugs. Therefore, the researchers are applying for a patent for this and plan to commercialize their technology.

**Figure 10:**
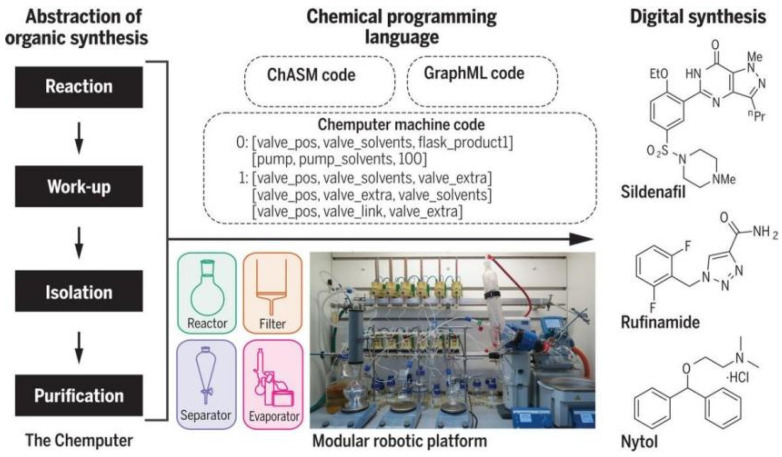
The Chemputer automated synthesis system and its application in drug synthesis. Reprinted with permission from ref. [58]. Copyright 2018, The American Association for the Advancement of Science.

Professor Lee Cronin et al. [58] designed and developed a decision-based automated program capable of training chemists to rely on vision for molecular assembly-related operations using a conductivity sensor that can outperform human vision (Figure 10). The automated process is implemented in a modular robotic platform that runs a chemical programming language to control the assembly of the molecules involved. Even users with no programming knowledge can easily code. As long as the necessary modules and drivers exist for Chemputer, users can run the published synthesis methods directly without reconfiguration. Using this automated synthesis system, the researchers were able to prepare three high-quality pharmaceutical compounds: diphenhydramine hydrochloride, lufenamide and sildenafil, without any human intervention, and the yields and purity of the three products were tested to be comparable to those of synthetic synthesis.

The Chemputer system consists of different modules, including a reaction module, an internal filtration module (which can be heated or cooled), an automated liquid–liquid separation module and a solvent evaporation module, which divides the different processes of chemical synthesis into icon-based modules. The entire chemical synthesis process requires not only Chemputer software but also some physical equipment: bottles of reagents, round-bottom flasks, filtration and liquid–liquid separation units, rotary evaporators, as well as pipes, valves and pumps for transporting chemicals. Researchers input synthesis methods and step-by-step instructions from the literature into steps suitable for automation and the synthesis steps are converted into digital codes that can be flexibly distributed between platforms without modification, thereby greatly improving reproducibility and the rate of synthesis of complex molecules. By developing control software to automatically clean and reuse the hardware modules in subsequent reaction steps, a multi-step synthesis process has been completed from individual unit operation, through to the combination of complete multi-step synthesis processes and ultimately for laboratory scale organic synthesis.

It is worth noting that, since its inception, the Chemputer still has many shortcomings to address. For example, the synthetic routes for all three drug molecules were relatively short and simple, overwhelmingly involving only extractive separations and it was difficult to achieve such high purity separations as described by the researchers in a step with many side reactions. In addition, analytical techniques such as LC-MS, NMR and IR are not mentioned in the text, indicating that these steps can only be repeated with the Chemputer system once the final optimization has been made.

### 2.4. Artificial Intelligence Cloud Lab

**Figure 11:**
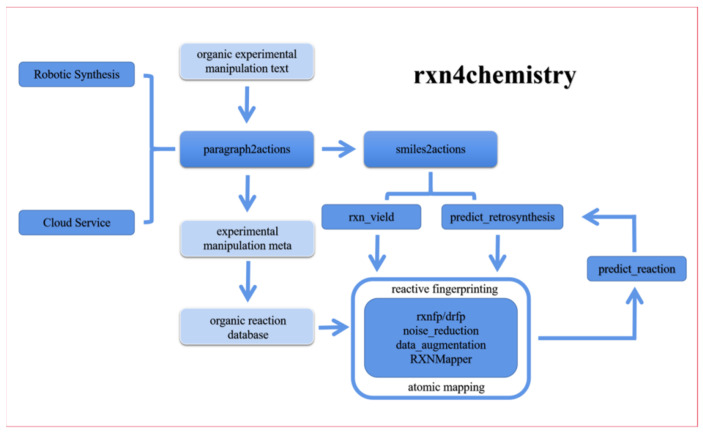
IBM RXN for Chemistry main work and its overall idea.

In 2020, IBM introduced a new industrial chemistry lab robot called RoboRXN [59] that applies artificial intelligence technology to organic synthesis, “the first remotely accessible, autonomous chemistry lab.” The RoboRXN project began in early 2018, the same year IBM released its junior version and offered its prediction services for free. Based on user feedback, RoboRXN has been continuously optimized and now has an accuracy rate of 90%, reportedly ranking as the top AI in the chemical reaction prediction category (Figure 11). Its biggest feature is the integration of artificial intelligence, cloud technology and experimental robot functions. All the chemist needs to do is log on to the website and draw the molecular structure formula, and RoboRXN will automatically give the synthesis route and send the instructions via cloud technology to the remote laboratory, where the robot inside will do all the work.

**Figure 12:**
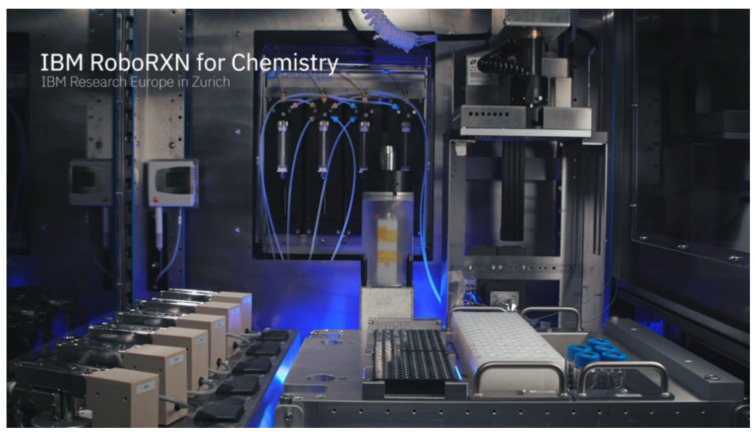
A corner of the RoboRXN Cloud Lab. Reprinted from ref. [59].

The main tasks of IBM RXN for Chemistry include: (1) Reading organic experimental operational texts from literature and patents and converting them into machine-readable operational elements. (2) Pre-processing reaction data (generation of reaction fingerprints, data enhancement, data noise reduction, atomic mapping of reaction materials to products). (3) Forward prediction of organic reactions. (4) Inverse synthesis reaction prediction. (5) Reaction yield prediction. (6) “Translation” of reaction SMILES into operational elements. These tasks constitute the cloud service of IBM RXN and also train a whole set of transformer-based artificial intelligence models, from reading the literature to learning the experimental operation, learning the organic reaction law, learning the forward reaction, reverse synthesis reaction and judging the reaction yield, and finally learning to design their own synthesis experiments for a new reaction—to a certain extent, to realize the “cloud-based services and AI-driven automated organic synthesis laboratory” concept (Figure 12). In addition, using the Transformer model, a top performer in the field of natural language processing, IBM researchers defined 24 experimental operations, extracted the reagents, dosages and conditions from the experimental operations and enabled the robot to read these operational actions, reagents, reagent dosages and reaction conditions to automate the synthesis.

### 2.5. Automated Synthesis Systems from Design to Synthesis

**Figure 13:**
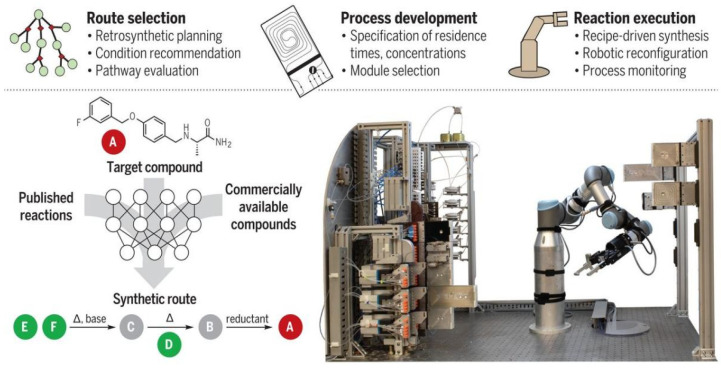
Automated synthesis system from design to synthesis. Reprinted with permission from ref. [60]. Copyright 2022, The American Association for the Advancement of Science.

In 2019, MIT researchers [60] made significant progress in two areas of algorithmic prediction of chemical reactions and automated laboratory equipment, using chemical reactions obtained from US patents and the Reaxys database to train artificial intelligence algorithms to design synthetic routes (including reaction conditions) for a given molecule and evaluate which route is best, based on the number of steps and expected yields (Figure 13). At the same time, the system also has a flexible robotic arm to automate all synthetic processes by connecting tubes supplying different reagents to flow chemistry modules such as reactors and membrane based separators to perform experiments. The robotic platform was used to successfully design and automate the synthesis of 15 chemical small molecules, including aspirin in 91% yield, (S)-warfarin in 78% yield with an enantiomeric ratio of 4.1:1 and two libraries of five drug-related molecules each, involving eight specific inverse synthetic routes and nine specific process configurations. The simplest of these synthesis processes takes two hours, while the more complex ones take about 68 h.

**Figure 14:**
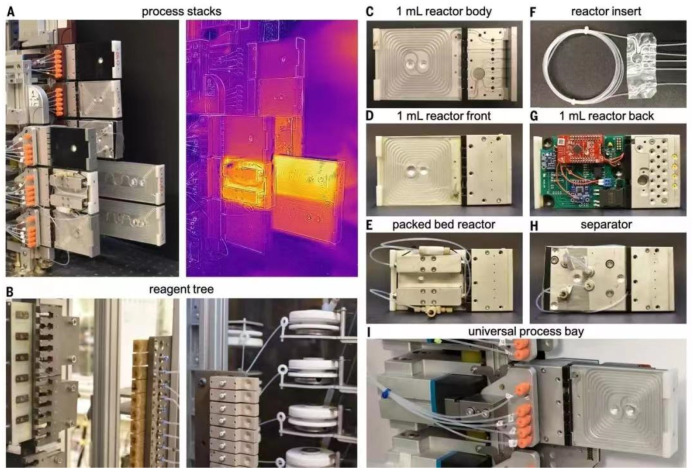
Component modules of the flow chemistry robotics platform: (**A**) process stack where modules are connected to the UPB to form a continuous flow path (**left**) and thermal image showing the heated reactor (**right**); (**B**) front view of the reagent tree and reagent manifold; (**C**) front view of the reactor body; (**D**) 1.0 mL reactor processing module; (**E**) double column packed bed reactor process module; (**F**) disposable PFA reactor insert; (**G**) integrated electronics on the back of the 1.0 mL reactor; (**H**) in-line membrane separator; (**I**) close-up of the UPB containing the 1.0 mL reactor. Reprinted with permission from ref. [60]. Copyright 2022, The American Association for the Advancement of Science.

The new system developed by MIT researchers combines three main steps: first, software guided by artificial intelligence suggests a pathway to synthesize the molecule, then professional chemists review the route and refine it into a chemical ‘recipe’ and finally the recipe will be sent to a robotic platform that automatically assembles the hardware and executes the reaction to build the molecule. The researchers have developed an open source software, ASKCOS, for the design of computer-aided inverse synthetic routes, using the Reaxys database and millions of reactions from the US Patent and Trademark Office for training, with the aim of applying inverse synthetic transformations by learning and determining the appropriate reaction conditions and evaluating the reactions. The algorithm used 12.5 million single-step reactions in Reaxys to attribute approximately 164,000 reliable rules. Then, a neural network model was trained to predict which of the 163,723 rules would best be translated and applied to the target molecular structure. In each case, the system asks whether any conditions exist that would transform a given reactant into the desired product. Ultimately the target compounds were traced back to small molecules that are readily and inexpensively available from suppliers such as Sigma-Aldrich. The system contains a process stack, reactor, separator and solvent tree as shown in the diagram. To execute the designed synthesis route, the robotic arm assembles the modular process units (reactor and separator) into a continuous flow route according to the configuration and the system starts synthesis by connecting the reagent lines and the computer-controlled pump to the reactor inlet via a fluid switching plate. After the specified synthesis time, the system flushes the line with a clean solvent and the robot arm disconnects the reagent line and moves the processing module to its proper storage location (Figure 14).

So far the system has only been able to use known reactions and there are a number of challenges: 1. The need for process intensification (e.g., reduction of reaction time), reduction of solid formation to avoid blocking and calculated predictions of solubility in non-aqueous solvents and at non-ambient temperatures remain difficult. 2. Predicting suitable purification methods is also very challenging, especially for non-column chromatography methods; 3. The examples of reactions shown in the article are single or two-step and the optimization of multi-step reactions can be complicated by the propagation of parameters. Overall, the integrated platform of CASP and mobile chemistry robots represents a milestone on the road to fully autonomous chemical synthesis.

Currently, multiple drugs are undergoing clinical trials in response to the new crown pneumonia (COVID-19) outbreak. There is no doubt that, in the face of possible drug shortages, the supply of drugs can only be accelerated by new methods of drug development. Thanks to the rapid development of artificial intelligence technology, chemists are ready for this “in advance”. In 2020, chemists Tim Cernak et al. [61] used Synthia artificial intelligence (AI) software to conduct a retrospective synthesis study of 12 investigational anti-COVID-19 drugs, finding new routes to synthesis for 11 of them using inexpensive and readily available raw materials while avoiding existing patents. In addition, the team has experimentally validated the feasibility and economics of the synthetic routes for two of the drugs, namely umifenovir and bromhexine. Synthia not only gave reaction routes, but also ranked the feasibility and economic viability of these routes. For the other candidate anti-neo-crown drugs, Synthia™ has also given predicted new routes. Although these routes have not been experimentally validated, such a diverse selection of starting materials also provides a new way of thinking about the raw materials for candidate anti-COVID-19 drugs.

### 2.6. Artificial Intelligent Fully Autonomous Mobile Robots

**Figure 15:**
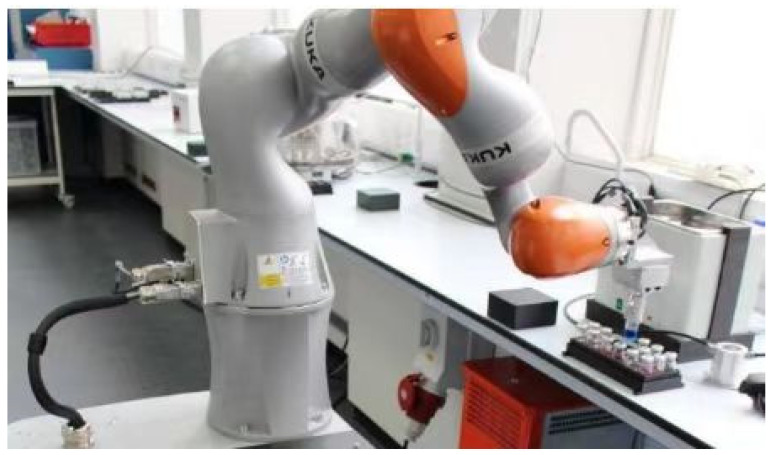
Fully autonomous mobile “Robot Chemist”, Reprinted with permission from ref. [62]. Copyright 2020, Spring Nature.

High-throughput chemical synthesis platforms are usually only capable of performing a single specific experimental task and do not allow the same freedom of movement in the laboratory as a researcher to perform a variety of experiments. Can “Robot Chemists” be made to break through this limitation? In 2020, Professor Andrew I. Cooper et al. [62] designed a mobile robot with a robotic arm to help with photocatalytic hydrolysis experiments, promoting the development of “robot chemists” (Figure 15). The idea was of a mobile robot consisting of a robotic arm and a movable platform, the main parts of which are made by KUKA Robotics, with laser scanning and haptic feedback for precise positioning by itself. The designed robot is approximately 1.75 m high, weighs approximately 430 kg and can operate continuously for 21.6 h under the condition of full charge. In addition, the authors have equipped the robot with a multi-purpose fixture that allows it to move freely around the laboratory like a laboratory technician and perform various operations in the laboratory independently, including solid-liquid drug weighing and use, dispensing, sample delivery and sample storage at the gas chromatography station. At the same time, the authors have modified the laboratory to build eight different workstations to meet the six processes required for the experiments. After 688 experiments, the robot found that the best catalyst formulation was a mixture of 6 mg NaOH, 200 mg L-cysteine, 7.5 mg sodium disilicate, 5 mg photocatalyst and 5 mL water, with a hydrogen production rate of 21.05 µmol h^−1^, which was six times higher than the starting conditions. At present, the method also has some limitations. For example, Bayesian optimisation is somewhat blind due to the assumption that all variables have the same initial importance. In addition, such a robotic search would not capture existing chemical knowledge, nor would it include theoretical or physical models and there would be no computational brain. Meanwhile, this system is currently not capable of generating and testing scientific hypotheses on its own. The “Robot Chemist” is able to conduct continuous and efficient chemical experiments in the laboratory as a home. It is expected to be an important development direction in the field of chemistry and engineering in the future, which can be analyzed and screened from multidimensional condition variables and big data. The “Robot Chemist” they have designed and the search strategy also have the potential to be used further. At the same time, the use of robots can free up researchers’ hands to a certain extent, freeing up more time for scientists to think creatively and promoting the development of related professions such as chemistry.

## 3. The Research Progress of Artificial Intelligence in the Field of Energetic Materials and Its Application Prospect in the Field of Energetic Materials Nitrification

At present, there are no literature reports on artificial intelligence in the field of propellants and explosives nitrification. Now the research in the field of propellants and explosives is mainly focused on algorithms and methods for machine-based structural search and performance enhancement of energetic material and the research is relatively scattered.

### 3.1. The Research Progress of Artificial Intelligence in the Field of Energetic Materials

#### 3.1.1. Advances in Artificial Intelligence Algorithms and Methods for Performance Enhancement of Energetic Materials

As early as 2005, N. Nariman-Zadeh’s research group proposed the evolutionary method network of generalized GMDH (group method of data handling) model design and successfully applied it to the modeling of complex propellants and the explosives forming process [63]. In this method, genetic algorithm (GA) and singular value decomposition (SVD) were used to optimize the connectivity configurations and coefficient values of GMDH type. Neural network was used to simulate the central deformation, circumferential strain and thickness strain of propellants and the explosives forming process. It was worth mentioning that this study proposed a new coding scheme that genetically designed generalized GMDH neural networks, whose connectivity configuration was not limited to adjacent layers. This generalization of the network topology provided an optimal network in terms of the number of hidden layers and/or neurons, resulting in polynomial expressions of the process dependent variables. It was also proved that the performance of GS-GMDH network is better than that of CS-GMDH network, so it was more suitable for the number of modeling input variables for small or large processes. In addition, there was also evidence that singular value decomposition (SVD) can be effectively embodied in GMDH- type networks by using the coefficient vectors of quadratic sub-expressions.

**Figure 16:**
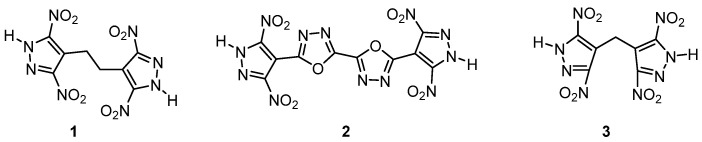
The structural formula for 1, 2 and 3. Adapted from ref. [64].

In 2020, Michael Gozin et al. introduced a new explosive at the molecular level that sacrificed “complex” bridge parts or “fused” annular arrangement structures [64]. The authors found that there was no direct correlation between the bond strength and thermal stability of energetic materials and the correlation between the two had not been reported before. The authors used generalized density functional theory (DFT) to characterize the molecular and crystal level data of 60 high energy materials (EMs) and the key descriptions that defined these characteristics correspond to the thermal stability of the compounds. The proposed descriptors for thermostable EMs were of a type of molecular design, location and type of the weakest bond in the energetic molecule, as well as crystal packing coefficient, specific ranges of oxygen balance, crystal lattice energy and Hirshfeld surface hydrogen bonding. On this basis, three new heat-resistant EMs were designed and synthesized, including the bridged 3, 5-dinitropyrazole parts 1, 2, 3 and the detonation performance was tested. The results showed that the initial decomposition temperature of 3 (below) with the best comprehensive properties was 341 ℃, the density was 1.865 g·cm^−3^, the calculated detonation velocity and the maximum detonation pressures were 8517 m·s^−1^ and 30.6 GPa, respectively (Figure 16). The author believed that the proposed molecular and crystal criteria had a certain reference value and indispensable assets for the design of heat-resistant explosives and a very high reference value for the design of heat-resistant energetic materials in the later stage.

In 2020, Michael Gozin et al. used supercomputers and the latest quantum computing strategies to perform high-throughput quantum calculations on 67 typical high-energy-density materials (HEDMs), faced with the problem that the gap between the explosion and stability of new HEDMs at regular intervals remained a formidable challenge [65]. Detonation properties, crystal stability and dozens of physical and chemical parameters were calculated for each compound and detailed comparisons were made with the reported data. Based on a large number of physicochemical statistical analysis data, the interspecific interaction in crystals was considered to be the main cause of the stability contradiction of HEDM properties. In view of this, the authors put forward the following suggestions for the design of new HEDM skeleton materials in the later stage: (1) molecular structure: bridge or non-planar heterocyclic/cage structure; (2) crystal structure: planar and cross-layered; (3) key parameters: nitrogen density > 0.6 g·cm^−3^, −40% < oxygen balance < 0%, filling coefficient > 73%, material density > 1.73 g·cm^−3^. To the best of our knowledge, the comprehensive and systematic strategies presented in this study will provide strong guidance for the crystal design of novel HEDM materials.

In 2022, Stephen Baek et al. reviewed approaches based on three major stages of material-by-design, namely representation learning of microstructure morphology (i.e., shape descriptors), optimization/design exploration and structure-property-performance (S-P-P) linkage estimation [66]. The EM-specific needs and challenges of AI/ML application level for various MbD problems were discussed. AI/ML for EM microstructure MBDS was still in its infancy, but showed great potential in terms of performance, efficiency and practicality, providing a perspective on the potential, practicality and effectiveness of achieving design materials for these methods. Finally, there was a gap between the AI/ML algorithms and computing power and the actual formulation and manufacturing of EM that needed to be closed before a data-driven MdB cycle can be implemented. The combination of anticipated material models, computational methods, software and hardware improvements and rapidly evolving AI/ML approaches was likely to make the silicon design of materials (including EM) a strong driver for the development of novel, precise and customized materials in the coming decades. The authors suggested a number of promising future research directions for EM materials-by-design, active learning, such as meta-learning, semi/weakly supervised learning and Bayesian learning, to bridge the gap between machine learning research and EM research.

It has been an eternal goal of military and civil fields to search for high energy compounds with excellent detonation performance and insensitivity to external stimuli [67,68,69]. In 2022, Bozhou Wang et al. conducted a detailed review of classical and new propellants and explosives groups and various propellants and explosives groups since 2010 [70]. In order to help design new energetic compounds of HEDMs in the future, better understanding is required of the structure–property relationships of energetic compounds. The authors summarized a series of strategies using big data, including: (i) creating more dynamic frameworks, (ii) introducing a large number of oxygen-rich explosive groups to improve oxygen balance, (iii) construct dense energy structures by introducing fragments with strong intramolecular and intermolecular interactions. The authors suggested that future HEDM synthesis requires the use of inexpensive raw materials and the easy and rapid synthesis of more stable target structures.

In 2023, Didier Mathieu et al. published an example of a deep learning method, compared it with “traditional” QSPR and semi-empirical methods to predict molecular properties and discussed energetic materials based on their inherent specificity [71]. It was proved that the latter was the domain knowledge and was not mandatory and the prediction model of rapid development had rationality and accuracy. This asset-over-functionality approach reduced engineering or time-consuming calculations because the performance of deep learning models was highly dependent on the quantity and quality of available databases. However, an unparalleled feature of deep learning technologies was that they can be used as new materials with target properties for generating models of design. Variational Auto Encoders were one such technique that allowed the establishment of a chemical space organized around a specific structure or property. In contrast, deep reinforcement learning offered the opportunity to incorporate empirical knowledge into the algorithm by defining chemically related behaviors. Finally, the author presented his own views on the most promising production strategies for the next silicon generation of new EM satisfying the performance/sensitivity trade-off in the future.

#### 3.1.2. Advances in Artificial Intelligence in Machine-Based Structure Search in Energetic Materials

In 2018, Daniel C. Elton et al. verified the concept that “machine learning technology can be used to predict CNOHF high-energy molecular properties from molecular structure” [72]. So far, however, candidates have used expensive quantum predictions to sift through the molecular simulations and thermochemical codes of high-energy materials. We focused on a small, diverse data set of 109 molecular structures distributed across 10 compound classes. Authors presented a comprehensive comparison of machine learning models and several molecular characterization methods, such as bond sums, coulomb matrices, custom descriptors, fingerprints and bond bags. The best feature was the sum of bonds (bond counts) and the best model was the Kernel Ridge regression model. Although the data set was small, we obtained acceptable errors and Pearson correlations for predicting detonation pressure, detonation velocity, explosion energy, heat of formation, density and other out-of-sample properties. By including another data set with ≈300 additional molecules in our training, we showed how the error can be pushed down, albeit more slowly with the number of molecules converging. The work paved the way for future applications of machine learning in this area, including automatic generation of cues and interpretation of machine learning models to gain new chemical insights.

In 2018, Brian C. Barnes et al. used machine learning techniques to rapidly predict detonation characteristics including detonation energy, detonation velocity and detonation pressure [73]. Property descriptors evaluated included morgan fingerprints, electronic state vectors, custom sum-on-key descriptors and coulomb matrices. The algorithms discussed include kernel ridge regression, least absolute shrinkage and selection operator (“LASSO”) regression, Gaussian process regression and multilayer perceptron (neural network). The effects of regularization, kernel selection and network were discussed in terms of parameters, dimension reduction, and so on. By using this method, authors successfully demonstrated the application characteristics of data driven and machine learning in predicting molecular detonation characteristics. In addition, the authors proposed that future work also included high energy material precursors for generating neural networks for the creation of novel high performance molecular recommendation networks and extended neural network predictive synthesis pathways. Future work should also include the creation of generative neural networks to suggest new high-performance molecules and the extension of neural network techniques to predict synthetic pathways for precursors of high-energy materials.

Predictive chemistry has become one of the most important research directions in machine learning. In recent years, more and more attention has been paid to using machine learning techniques to predict the properties of energetic materials. In view of this, in 2022, Yi Wang et al. reviewed the recent research progress in using machine learning to predict the properties of energetic compounds (e.g., density, the heat of the explosion, detonation velocity, sensitivity, enthalpy of formation and decomposition temperature) [74]. The results showed that machine learning had made remarkable progress in predicting the properties of energetic materials, but it had not been able to obtain satisfactory results in predicting the precision decomposition, melting temperature, sensitivity and other properties of compounds based on electronic structure or molecular dynamics. The main reasons were: (1) the noise in the data caused by the passing experimental conditions, (2) the quantum scale of continuum mechanics with intrinsic multi-scale properties obtained, (3) the coupling process behind these properties of complex thermochemistry. Predictably, these problems were difficult to solve in the short term. Possible solutions included more complex model architectures trained on big data, such as GNNS, and more complete and physically meaningful representations of molecules. However, these approaches often mean increased model complexity and require large amounts of data to avoid overfitting. Lack of data was another difficulty limiting machine applications due to military applications, learning and danger in the field of energetic materials. Therefore, there was an urgent need to establish a database of standard energetic materials, which may require collaboration of the entire energetic material community. To sum up, at present, the research of machine learning technology in the field of energetic materials is still in its infancy.

The direct analysis of the three-dimensional electronic structure of molecules has always been a technical problem in the field of energetic materials artificial intelligence. In 2020, Alex D. Casey et al. developed a convolutional neural network to directly analyze the three-dimensional electronic structure of molecules using space point data to describe the charge density and static potential expressed as the 4D tensor [75]. This approach effectively bypassed the need to construct complex representations, or descriptors, of molecules. This was helpful because accurate machine learning models were only as good as input representations. The experimental results showed that the 3D convolutional neural network can effectively analyze the electron charge density and electrostatic potential of molecules, so as to predict the material properties with high precision, without the need to make molecular descriptors manually. This model was superior to the random forest model tuned using the ECFP4 descriptor. The model achieved excellent generalization errors even when making predictions for molecules with different structures, as observed for stent-based splitting. In addition, the model can be coupled with other machines that can estimate the electronic structure of learning models so that physics-based workflows completely bypassed enhanced material screening and accelerated discovery. Finally, the work of quantifying the uncertainty of model prediction would greatly improve the practicality of the model. This work was also the first machine learning of molecular properties using complete 3D electronic structures.

In 2022, Dylan Walters et al. developed a machine learning (ML) model that connected the microstructure details of energetic material formulations to guide the discovery of properties of new explosive formulations [76]. The robustness of the ML algorithm developed by Sen and Rai was investigated by using distinct microstructural features (characteristic of VSD) and calculating boot performance indicators. The results showed that the tool was indeed robust in predicting the initiation performance of oil and gas wells. In addition, it correctly identified the most important information factors. However, the algorithm did not recognize more nuanced effects of VSD characteristics, having a weaker effect that might be detectable by an experienced human observer. However, overall, this was a very promising approach, because a small team of experts with limited resources can produce a widely available tool for optimizing propellants and explosives formulations to meet specific performance specifications. In addition, it was worth mentioning that the authors intended to extend the tool to a wider range of areas by expanding the training data to include chemical dynamics factors beyond the microscopic structural details considered here to identify propellants and explosives.

The realization of accurate molecular design and efficient green synthesis has been one of the main research directions of EMs. The research and development of high-performance energetic materials has required a process of trial and error and required a long research and development cycle, high research and development costs. In recent years, machine learning (ML) has become a supplementary and auxiliary experimental research tool, which can perfectly solve the problems of long period cycle and high research and development costs. It could be used to predict and design EM with novel structure and has made great research progress in recent years. In view of this, in 2023, Ruiqi Shen et al. reviewed the key processes of ML method discovery and prediction of EMs including data preparation, feature extraction, model construction and model performance evaluation [77]. The main ideas and basic steps of applied machine learning methods were analyzed and summarized. The application of machine learning in electronic material performance prediction and reverse material design was further summarized. Finally, the challenges existing in the further application of ML method and the strategies to deal with them were proposed. The author pointed out that. although machine learning methods had been successfully applied in a large number of cases, they were still in the early stage of development to a large extent, and believed that machine learning would play an increasingly important role in accelerating the development of artificial intelligence in the foreseeable future.

### 3.2. The Inspiration of Artificial Intelligence in Development in the Drug Field on the Nitrification of Energetic Materials

From the above examples, AI synthesis currently has made a major breakthrough in the field of drug development, but there is no report on AI nitro synthesis of energetic materials at home and abroad. Compared with drug synthesis, nitro synthesis of energetic materials exhibits its own characteristics, mainly as follows. 

(1) There is little basic data accumulated on the synthesis process of energetic materials, making it difficult to employ the efficacy of big data technology and artificial intelligence algorithms. The design of drug molecule synthetic routes usually requires more than 10 million reaction data for training to achieve high prediction accuracy. 

(2) It is relatively difficult to synthesize energetic materials by nitrification, which usually requires demanding reaction conditions and tedious pre- and post-treatment processes and high manual intervention. The synthesis of energy-containing materials is generally associated with significant exothermic and corrosive characteristics, requiring customized synthesis plants. 

(3) Therefore, the direct transplantation of intelligent synthesis techniques in the pharmaceutical field is expected to have difficulty in achieving ideal results in the synthesis of energetic materials by nitrification and research needs to be carried out on various reaction systems, taking into account the nitrification characteristics of energy-containing materials.

Based on the above-mentioned nitro-synthesis characteristics of energetic materials, the design principle of the artificially intelligent synthetic nitro-synthesis reaction relies on continuous flow and intelligent robotics designed to simulate the workflow of artificial organic synthesis. The artificially intelligent robot resembles a mobile robot with a robotic arm similar in size to human beings, which can operate a variety of instruments and equipment in the laboratory. The robots are positioned by laser scanning and touch feedback and move freely in the laboratory or workshop to independently perform various tasks in experiments, such as weighing solids, dispensing liquids, degassing vessels, reaction processing and quantifying nitrification reaction products. The robot system consists of four main parts, including a controller system, robot body, actuator and power system, and end-effector. For example, strategies involving modern data analysis methods and advanced parameter sets can be used in the nitrification reaction of energetic materials, in which an integrated set of descriptors obtained from quantitative constitutive relationships (QSAR), molecular mechanics (MM) and density flooding theory (DFT) is associated with a relatively large library of nitration reaction outputs acquired by multiple literature sources through data mining [78,79]. General relationships between reactions can be established by combining suitable data organization and trend analysis techniques. Finally, the prediction of new nitrification reaction types is validated by statistical modeling. The first step is to use the already commonly used and standardized operations of synthetic chemical units (e.g., separation, filtration, evaporation, heating, cooling, stirring, etc.) to achieve automation in a uniform way.

It is crucial to define a set of unit operations to achieve this, which can be described as an explicit chemical language that is interpreted and executed by standard automated chemical hardware. The standard hardware required for the nitrification of energetic compounds is clearly defined and the unit operations performed by these hardware (dosing, stirring and heating) can all be conducted independently and are easily combined. The highly modular approach makes the architecture easily scalable and changes to one module do not affect other parts of the system. The feature also means that the operation of each unit can be easily controlled by code, making the system programmable. The first step of automated synthesis is to analyze the basic unit operations required for the reaction. Once these unit operations are identified, each unit operation can be connected to the corresponding hardware module via a software interface based on given graphics files. After the preparation, the computer can automatically perform the appropriate action for each reaction. The system exhibits the following features [80,81,82]: (1) High efficiency: High-throughput screening, optimization and synthesis abilities, capable of carrying out hundreds to thousands of nitrification chemical reactions in a single day. (2) Safety: All experimental operations involved are performed automatically by the machine without human involvement on site. (3) Intelligence: The machine decides the optimal reaction planning for the next step using big data and the results of the previous step of the experiments.

### 3.3. Perspectives on the Application of Artificial Intelligence in the Field of Nitrification of Energetic Materials

At present, Europe and America are in the leading position in technology in the field of intelligent synthesis and have developed corresponding design algorithms, programming languages and automatic devices. They initially opened up the logical chains from route design to automatic synthesis and obtained typical verification in drug synthesis. Other countries are in a follower position and have certain capabilities in equipment development, but there is still a gap in algorithm development and the logic chains have not yet been broken. Many research institutions and drug companies have started to enter the field. For example, the Magenta Technology Company is committed to establishing the first self-researched intelligent automated biological laboratory in China. Based on robotics, automation, software control, pipetting equipment, testing equipment, data processing and other technologies, we developed our universal laboratory automation platform system. The system takes the robot arm as the center and connects to various types of laboratory instruments and equipment to realize fully unattended and fully automated experimental operations, such as pipetting, centrifugation, shaking and testing.

AI has been included in many countries’ national strategy and is representative of new technologies and productivity. It is imperative to push into AI in the field of nitrification of energetic compounds. The integration of artificial intelligence synthesis with the nitrification reactions of energetic compounds contributed to the advancement of nitrification technology by enabling the instruments and software the ability to analyze and process the nitrification reactions of energetic compounds autonomously. AI reduces the cost of nitrification operations, improves operational efficiency, significantly reduces the number of personnel working on site, enables precise operation and improves intrinsic safety. The intelligent operation of AI even leads to pioneering changes in nitrification technology in some cases, while ensuring safety, which is of great significance for the implementation of “human-machine isolation” measures for the nitrification of energetic compounds.

Although there are no reports on AI nitro synthesis at home or abroad, it is foreseeable that the world’s military powers are bound to take major actions in this field in the near future. Once a breakthrough is made in the artificially intelligent nitration synthesis system, it will subvert the current research and development mode of new materials for fire explosives and enable the efficient, safe and intelligent automatic creation of new energetic materials, thereby rapidly meeting the needs of new conceptual modes of warfare for energetic materials with specific properties.

Undoubtedly, the strategy of combining artificially intelligent synthesis with nitrification reactions starts from the process. Complete human–machine isolation is achieved by using the artificially intelligent synthesis nitrification process, which suppresses the possibility of accidents at the source and facilitates a new digital era in the nitrification of hazardous energetic compounds. However, there are still two aspects that deserve some thought. 

First, the problem of cost deserves our deep consideration. Although AI is far more cost-effective than manual operations in the long run, the high upfront investment and long payback period make it very difficult to start. The most common problems with high-throughput instruments running AI that occurs are not major machine failures, but are more minor. What goes wrong is usually not computer hardware, but code. Every use of high-throughput equipment involving AI requires a professional, first to carefully design the entire process and then to write the code, script, or process to ensure that there are no problems at every step or that there are solutions when problems occur. Although the current AI experimental technology is facing many challenges, its powerful capabilities and great potential will effectively promote the development and upgrading of the nitrification process in the field of propellants and explosives. 

Second, nitrification and synthesis of energetic materials is relatively difficult, which usually requires harsh reaction conditions and tedious pre- and post-reaction processing along with more manual intervention. In addition, the synthesis of energetic materials is usually accompanied by a large amount of heat release, corrosion and other characteristics, so the synthesis device needs to be customized. Therefore, it can be predicted that direct transplantation of intelligent synthesis technology in the field of drugs will find it difficult to achieve a good effect in the nitrification synthesis of energetic materials. It is necessary to combine the characteristics of nitrification of energetic materials and carry out corresponding research for different reaction systems.

While the nitrification and synthesis of AI energetic materials will go a long way, the energetic materials industry’s initiative in embracing digitalization based on new technologies is the trend of the future. The introduction of artificial intelligence technology into the field of energetic materials will be a most promising approach to achieve high technology, high quality and high efficiency production of energetic materials.

## Figures and Tables

**Table 1:** Summary of representative work in the field of automated and intelligent synthesis.

Year	Institution	Name Of The Platform	Features
2015	Merck	High throughput chemical reaction screening platform	Screening of substrates and reaction conditions for Buchwald-Hartwig coupling reactions at the nanomolar level is possible, with 1536 reactions completed per day.
2018	Pfizer	High throughput chemical reaction screening platform	Screening of over 1500 nano-molar scale Suzuki-Miyaura coupling reactions in 1 day; synthesis of hundreds of micro-molar scale products.
2018	University of Glasgow, USA	Chemputer	(1) standardization of language and modularity of equipment, resulting in the preparation of three high-quality medicinal compounds without human intervention, with yields and purity comparable to those of artificial synthesis. (2) The ability to accurately predict the outcome of chemical reactions and to "think" independently after completing experiments, allowing for the independent exploration of new chemical reactions and molecules. (3) Automated synthesis "from literature to compound".
2019	MIT	-	The synthetic route design software ASKCOS was developed to automate the synthesis of 15 drug molecules based on the designed routes.
2020	IBM	RoboRXN	The three functions of artificial intelligence, cloud technology and experimental robotics have been integrated to develop the synthesis design system RXN for cloud-controlled automated synthesis based on designed synthesis routes.
2020	University of Liverpool	Mobile chemist	Freedom of movement within the laboratory to perform various tasks in experiments independently. The analysis of 10 dimensional variables in more than 98 million alternative experiments and the autonomous adjustment of the catalyst composition led to the discovery of a photocatalyst system with six times higher activity than the initial catalyst.

## Data Availability

Not applicable.
